# An Event based Body Coupled Transdural Telemetry for Intracortical Brain Computer Interfaces

**DOI:** 10.1038/s44172-026-00735-z

**Published:** 2026-09-01

**Authors:** Chengyao Shi, Laura Nuttin, Zhenyu Gao, Yuming He, Pietro Russo, Hua-Peng Liaw, Marios Gourdouparis, Dennis Lambrechts, Guido Dolmans, Yao-Hong Liu

**Affiliations:** 1 imec - Netherlands, AE Eindhoven, The Netherlands; 2 Eindhoven University of Technology, Eindhoven, The Netherlands; 3 imec, Leuven, Belgium; 4 Department of Neuroscience, Erasmus MC, GD Rotterdam, The Netherlands; 5 Department of Neurosurgery, Erasmus MC, GD Rotterdam, The Netherlands

**Keywords:** Electrical and electronic engineering, Biomedical engineering, Therapeutics, Neuroscience

## Abstract

Intracortical brain-computer interfaces (iBCIs) hold promise for restoring motor, sensory, and cognitive functions, including applications in paralysis treatment and speech decoding. High-density microelectrode arrays (MEAs) provide fine spatial and temporal discrimination of neural activity, but the considerable upsurge in data generation poses challenges for wireless transmission from miniaturized implants due to constraints on power, bandwidth, heat dissipation, and device size. To address these constraints, we propose a two-stage wireless iBCI architecture comprising a transdural galvanic-coupled body channel communication (BCC) link from a free-floating MEA to an intracranial unit, followed by a transcutaneous link to an external unit. This study focuses on the transdural BCC telemetry system, which provides compact, wideband, and energy-efficient data transmission. Phantom tests, and ex vivo experiments using a human cadaveric head specimen validate the system, demonstrating wireless transmission up to 500 Mbps with 20% duty cycling and bit error rates below 10⁻⁵. The system incorporates the send-on-delta encoder (SODA), achieving up to 11.4× data compression and reducing thermal load for meeting the safety guidelines. Safety is further examined using brain-on-a-chip models, which demonstrate that the system does not evoke unintended neural activity, supporting the platform’s long-term viability for high-resolution iBCIs.

## Abstract (web summary)

Chengyao Shi and colleagues demonstrate an event-based compression and body channel communication for energy-efficient and high-bandwidth intracortical brain-computer interfaces (iBCIs). This framework lays the ground for transdural communication for distributed and free-floating brain implants.

## Introduction

The demand for effective treatments for neurological disorders is expected to grow significantly in the coming decades. Neural interface technologies, including brain-computer interfaces (BCIs), are emerging as a promising technology for restoring motor, sensory, or even cognitive functions, such as motor intent prediction for paralysis^1^ or speech decoding^2^. Advancements in these technologies are crucial for enhancing the quality of life and reducing the burden on healthcare systems.

Intracortical brain-computer interfaces (iBCIs) utilize penetrating neural probes with microelectrode arrays (MEAs) to record extracellular neuronal activities, including local field potentials and action potentials (i.e., spikes), with exceptional spatial and temporal resolutions. The recent development of high-density MEAs (e.g., silicon probe^3,4^ or polymer probe^5^ with up to 1000 electrodes), has significantly increased the spatial resolution of neural recordings to just 10’s of µm^6^. Assuming each recording channel is sampled with a 10-bit ADC at 30 ksps, the total amount of data that needs to be transferred can reach 300 Mbps when the MEA has 1000 channels^4^. This substantial data throughput presents a major challenge for the implant’s telemetry system, which must wirelessly transmit neural data to an external processor without creating bandwidth bottlenecks, all while remaining compact to fit within a constrained implant volume.

Recent advancements in burr-hole craniotomy provide a minimally invasive access through openings less than 10 mm in diameter, enabling faster healing compared to traditional large-area craniotomies^7^. This advancement requires miniaturizing implantable devices, especially the telemetry system, which represents a key bottleneck in overall device footprint scaling due to constraints imposed by antenna size. Compact designs demand careful thermal management, as excessive heat can harm brain tissue. To reduce thermal risk, the telemetry module (which is typically the most power-consuming part of the implant) must consume within a few milliwatts of power^8^.

**Fig. 1:**
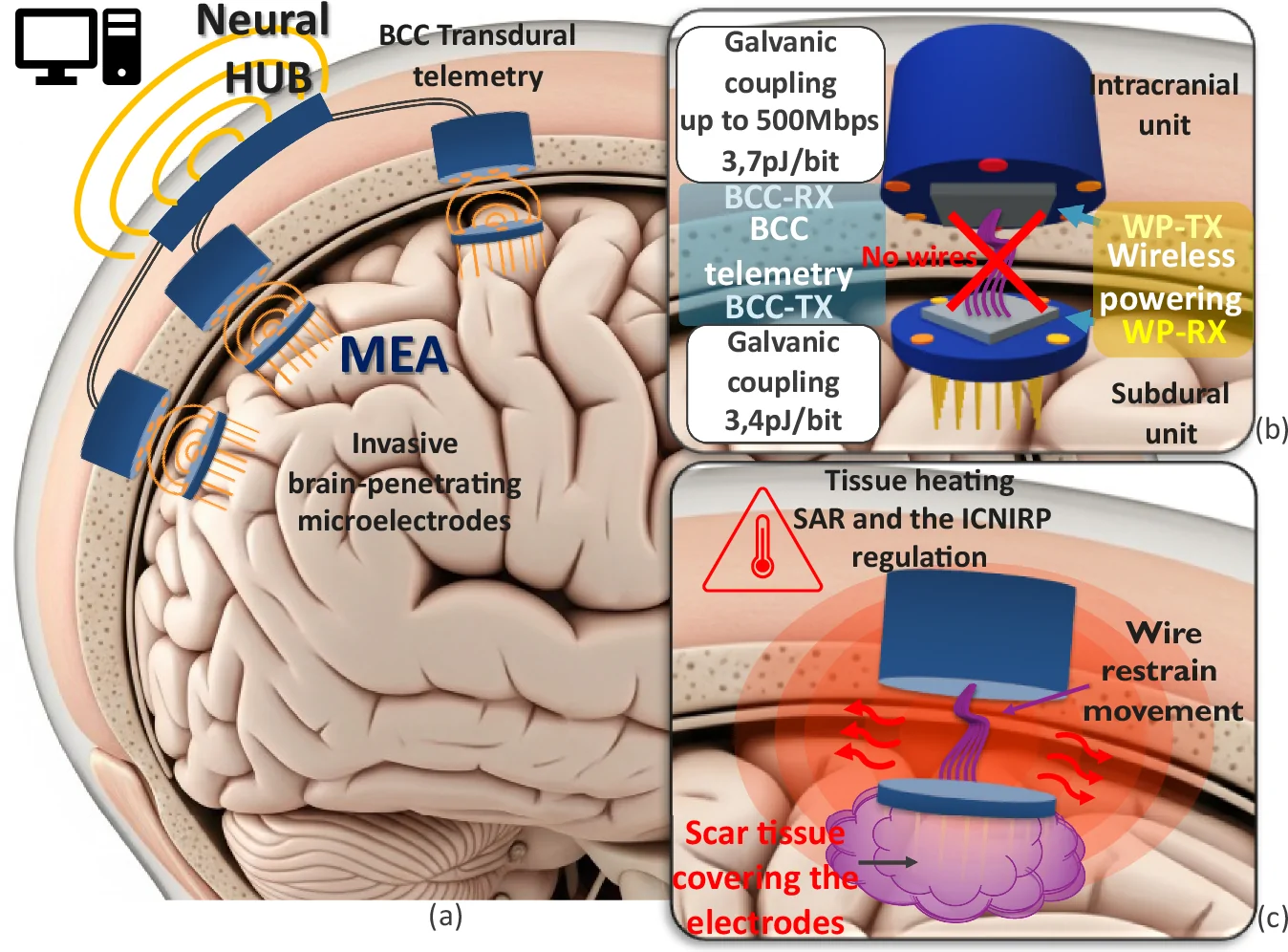
Proposed intracortical brain-computer interfaces (iBCIs). **a** The proposed two-stage wireless iBCI architecture features a transdural galvanic-coupled BCC link between a free-floating MEA and an intracranial unit, as well as a neural hub that aggregates and relays data from multiple MEAs for further processing. **b** The proposed transdural BCC communication system comprises a subdural unit integrated with the MEA and an intracranial receiving unit positioned on top of the dura mater. An wireless power (WP) delivery is envisioned in future implementation but is outside the scope of this work. **c** The formation of scar tissue is often attributed to the presence of wired connections, as the MEA is unable to accommodate the brain’s natural micromovements. Additionally, the proposed system is designed for low-power operation and compliance with relevant exposure constraints.

Motor, sensory, and cognitive functions involve distributed brain networks. As the brain integrates multisensory inputs to guide decision-making, multi-site recordings using distributed neural implants, i.e., distributed iBCIs, as conceptually illustrated in Fig. 1a, can provide valuable insights into these processes^9,10^. Multiple distributed MEA implants record neural activity, which is then collected and transmitted by a central neural hub.

Tethered distributed iBCI systems, where the intracortical MEA is connected via cable to a telemetry module on or above the skull, restrict relative motion between the MEA and the brain, as illustrated in Fig. 1c. This mechanical constraint prevents the MEA from accommodating natural brain movement, leading to micromotion-induced tissue damage, scar formation, and progressive signal degradation^11^.

To address these limitations, we propose a distributed iBCI architecture comprising a two-stage communication system: the first stage a “transdural” link between a free-floating subdural unit and an intracranial unit. The subdural unit is designed to integrate a microelectrode array (MEA), the SODA module, and the proposed body channel communication (BCC) transmitter. It is intended to operate as a free-floating module, without any physical connection to the BCC receiver. This is also the condition when we perform ex-vivo validation with the human cadaver. The intracranial unit incorporates the BCC receiver and is anchored to the skull at the burr hole created during surgery.

Then, it is followed by a second stage of a transcutaneous link in which a neural hub deserializes and relays the collected data to an external device (e.g., a desktop) for further processing, using wireless technology such as impulse radio ultra-wideband (IR-UWB)^12^, as shown in Fig. 1a.

This work focuses on the demonstration of the first stage of the proposed system: establishing a minimally invasive transdural connection that could potentially minimize scar tissue formation in the brain. We introduce a miniature and wideband wireless telemetry system designed to transmit neural signals across the dura mater, the brain’s protective membrane. This transdural communication is crucial for minimizing invasiveness by enabling a “free-floating” MEA design and by allowing the sealing of the dura layer, both of which avoid scarring. This approach is highly preferable for clinical applications^13–15^.

However, the strict constraints on the implant size, power consumption, and data rate in BCI applications present significant challenges for transdural wireless communication. Telemetry methods based on far-field electromagnetic radiation encounter a trade-off between antenna size and bandwidth. To achieve optimal radiation efficiency, the antenna size must correspond to the signal wavelength. For compatibility with burr-hole craniotomy, the antenna dimension should be in the order of a few centimetres. Although higher transmission frequencies ( > 1 GHz) reduce antenna size, tissue attenuation increases with frequency, leading to greater path loss due to the body’s shadowing effect^16,17^.

Inductive-based telemetry is widely used for implantable devices where data rate and size requirements are less stringent, such as in pacemaker applications. However, due to a high quality factor resonator required for efficient magnetic induction, inductive coupling does not provide sufficient bandwidth for the high data rate transmission needed for BCI applications. Additionally, coil miniaturization presents challenges because channel path loss and misalignment tolerance are dependent on the coil diameter^18–21^.

Telemetry based on optical communication has been shown to achieve high data rates while maintaining a small footprint. However, the link stability is highly sensitive to misalignments, and path loss varies depending on the tissue properties^22^.

Galvanic coupled BCC has been demonstrated to meet the stringent requirements for the transdural communication in distributed iBCI, demonstrating excellent scalability to multi-node MEA systems with spatial node distribution as small as 10 mm in separation distance^23^. BCC utilizes the human body as a transmission medium, with signals transmitted through biological tissue as electrical currents. Its high energy efficiency, scalability, high data rates ( > 500 Mbps), and secure transmission capabilities make BCC a promising candidate for the distributed iBCI applications. It achieves high data throughput and maintains a small footprint below 100 mm², all while ensuring low power consumption to below 1 mW. Finite element method (FEM) simulations conducted using COMSOL on a human head model were validated through both tissue-mimicking phantom studies and experiments involving real biological tissue.

As intracortical neural recording systems scale, data transfer and power demands grow. While many on-implant lossy data compression methods (e.g., spike sorting) are investigated^24^, the ability to reconstruct spike waveforms with high fidelity remains highly preferred by neuroscientists or clinicians for diagnostic or implant optimization reasons, and research purposes. As such, most implantable neural sensors reported in the literature for recording intracortical extracellular single-unit neural activities (SUA) from the brain prefer loss-less or low-loss data compression before wireless transmission^25,26^.

Neurons typically fire action potentials sparsely over time, with each action potential being brief in duration of less than 1 millisecond, and exhibiting a firing rate in the range of a few to 10’s of Hz. Using a conventional Nyquist-rate ADC would require a sampling rate of 10’s of kHz, which action potentials only occupy a small proportion of the data. Send-on-delta encoder (SODA), as proposed in^27^, it implements a Δ modulator and an event-based packetizer that transmits data packets only during neural activity, i.e., action potentials (or spikes), remaining silent otherwise. This approach achieves an overall data compression of 5–10×, allowing the BCC to be duty cycled to 10–20%.

As implants become smaller yet supporting more channels, thermal safety, and electromagnetic exposure, as regulated by the specific absorption rate (SAR) and the ICNIRP guidelines^28^, have become a critical constraint on the implant telemetry system. Duty-cycled transmission enables a safer implementation of telemetry by reducing the average transmission time and, therefore, lowering the power transmitted. Integrating the SODA with the transdural BCC is essential to ensure the operational safety of BCC telemetry while maintaining sufficient data fidelity during the limited active periods defined by the system’s duty cycle. We implemented a compact and battery-powered transdural telemetry system for the purpose of this validation. For future deployment, integrating wireless powering delivery strategies with a miniature form factor will be essential to enable long-term implantation. Notably, transdural wireless power transfer based on ultrasound technology has been demonstrated as an efficient approach for brain implant applications^29,30^.

This validation comprises three main sections. First, we present an integrated wireless telemetry system designed to meet the stringent requirements of distributed iBCIs, leveraging BCC galvanic coupling for its high bandwidth, efficiency, and scalability. Second, we assess the safety of BCC by investigating its potential to induce neural stimulation and demonstrating through experimentation that the implemented safety measures prevent such unintended effects. To the best of the authors’ knowledge, this represents the first known investigation of its kind. Finally, the system’s feasibility was validated through implantation in a human cadaveric head specimen, achieving wireless data transmission up to 500 Mbps (Manchester coded) and a bit error rate (BER) of <10^-5^.

## Results

### Event-based transdural BCC system design

**Fig. 2:**
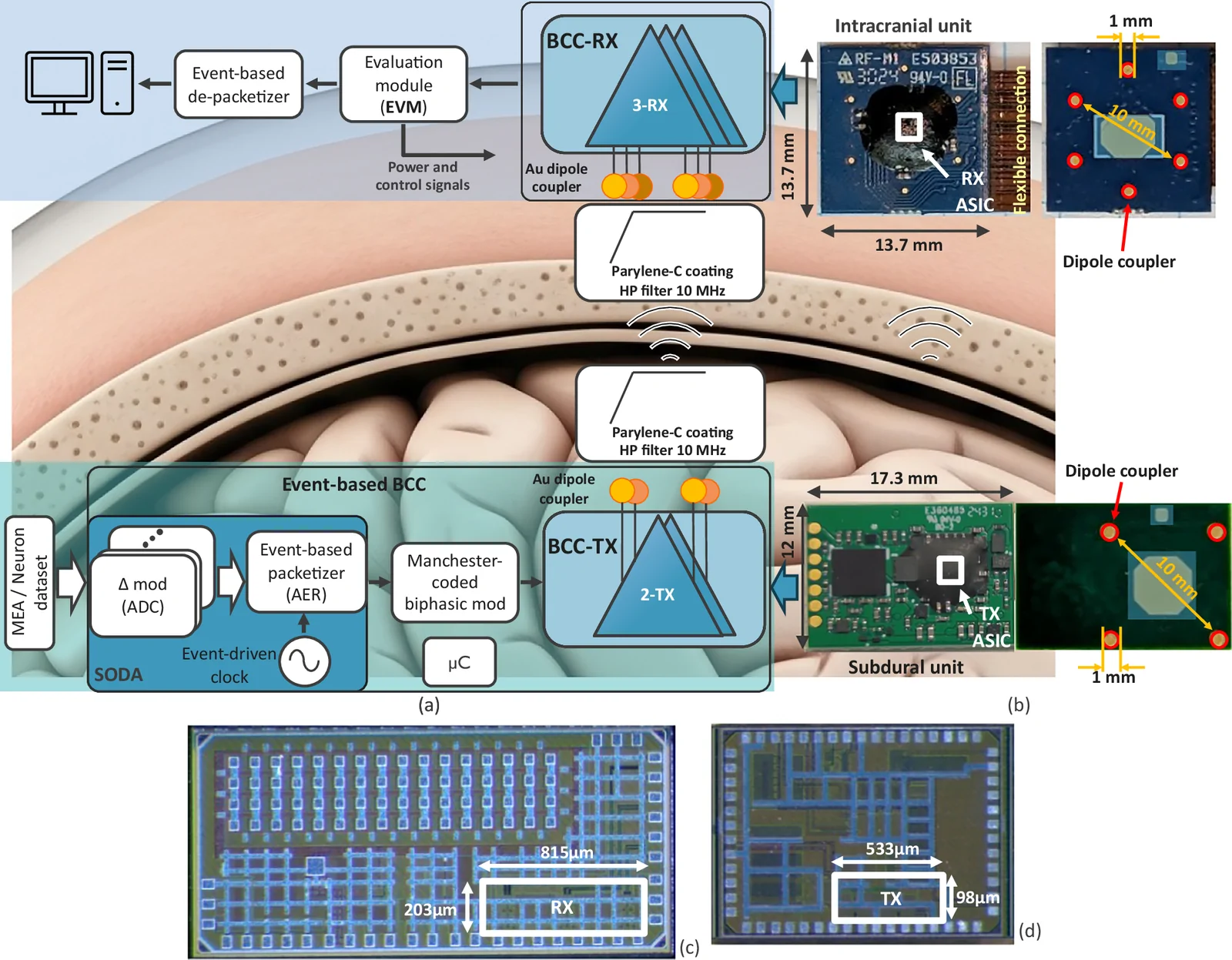
Transdural galvanic-coupled BCC link. **a** The block diagrams of the subdural and intracranial units. Both units are coated with Parylene-C, which provides hermetic sealing and functions as a high-pass filter with a cutoff frequency of approximately 10 MHz. The subdural unit interfaces directly with the MEA or a neuron dataset. The intracranial unit communicates with an evaluation module (EVM), which receives and deserializes the transmitted data for further processing. **b** Pictures of the subdural unit and the intracranial units. **c** The micrograph of the fabricated receiver ASIC. **d** The micrograph of the fabricated transmitter ASIC.

As illustrated in Fig. 2, the proposed telemetry system consists of two units. The first unit is the subdural transmitter, which samples the analog neural spike data from an MEA and sensor readout, compresses them via SODA, and transmits the data using BCC. The subdural unit is free-floating^31^ and constrained in size to ensure safe and stable implantation beneath the skull and dura. The second unit is the intracranial receiver, which receives data from the subdural transmitter. The design of the two modules is described in^23^.

An evaluation module (EVM) is implemented for testing purposes, to supply power and control signals to the BCC receiver application-specific integrated circuit (ASIC), and mimics the future neural hub. Positioned over a cranial aperture, the intracranial unit is less size-constrained, allowing for more design flexibility.

The subdural unit integrates a microcontroller (µC) for providing the configuration for the ASIC. For testing purposes, synthetic data is generated on-chip to simulate the MEA recording. With a power consumption of 2.4 mW, the subdural unit enabled continuous data transmission for approximately one hour during the experiment on a human cadaveric head specimen, using a button cell battery to power the subdural unit for experimental purposes.

On the receiving end, the intracranial unit receives the signal propagated through the biological tissue, amplifies it, and relays it to the EVM. For this validation, the BCC receiver prototype is implemented on a flexible-rigid printed circuit board (PCB), which facilitates measurements and connection to the EVM. The ASIC is mounted on the rigid portion of the PCB and aligned with the dipole coupler (a pair of coupling electrodes for the BCC communication), while the flexible section enables easier integration with the curved geometry of the biological sample. The EVM supplies power and control signals to the receiver ASIC on the intracranial unit.

### Biocompatible coating

We use Parylene-C through atomic vapour deposition coating to hermetically seal the devices throughout the duration of this study. The Parylene-C layer forms a moisture barrier capable of withstanding exposure to bodily fluids for the duration of the experiment, providing electrical isolation and preventing direct metal-to-tissue contact. Previous studies have indicated that Parylene-C is a valid substrate for the culture of neuronal networks for a duration exceeding three weeks^32,33^, and is found to be capable of providing the electrical insulation and encapsulation required for neural interface devices for more than one year^34^.

Moreover, the coating introduces a series capacitance at the dipole–tissue interface, forming an additional high-pass filter, with the corner frequency tunable by adjusting the coating thickness. In our application, the coating was designed to provide a corner frequency of ~10 MHz, which was selected based on the desired signal characteristics and the requirement to attenuate unwanted low-frequency components. The subdural and intracranial units were encapsulated in a 0.6-µm layer of Parylene-C, corresponding to a high-pass filter corner of ~10 MHz.

### Charge balance with a safety window

It is well established in the literature that neurostimulation devices must maintain charge neutrality between the injected charge and the charge sink to ensure safety^35,36^. Although galvanic BCC and neurostimulation devices are designed for different purposes and operate at different power levels, both induce ionic currents in biological tissue during operation. Therefore, safety principles derived from neurostimulation systems should be considered when evaluating galvanic BCC systems.

Research has shown that when electrical charge imbalances exceed a defined threshold, known as the “safety window”^35,37,38^, the resulting excess charge can harm tissue and damage electrodes due to the faradaic effect, an electrolysis process. Various techniques have been established to achieve net charge neutrality and thus prevent tissue damage. These techniques can be categorized into two types: active charge balancing and passive charge balancing.

Active charge-balancing methods actively adjust the injected electric current to achieve charge neutrality, often by modulating either the amplitude or the duration of the anodic phase of stimulation. Other active techniques involve additional injections of charges through impulse signals (pulse insertion) or an offset signal (offset regulation) once both cathodic and anodic phases have been completed in order to reach charge neutrality^35,39–43^.

In contrast, passive charge-balancing techniques rely on the natural redistribution of accumulated charges. A mechanism short-circuits the electrodes to redistribute the accumulated charges and reach charge neutrality^11^. An alternative passive method consists of blocking the DC components of the signal through a series of capacitors^44^.

The BCC telemetry system developed in^23^ employs two primary techniques to achieve charge balance. First, the data is encoded with the Manchester coding scheme to ensure a balance of ones and zeros, combined with biphasic pulse modulation, this approach ensures communication with no DC components. Second, a charge redistribution circuit integrated within the BCC driver short-circuits the dipole coupler at the end of each transmission packet, redistributing any residual charge that may accumulate due to circuit mismatches (PVT). The activation of this charge redistribution circuit is enabled through the duty cycling of the data transmission. Additionally, a thin Parylene-C coating provides extra series capacitance, further blocking low-frequency signal components.

Another important aspect of BCC communication is whether the electrodes can induce tissue damage. Tissue damage caused by electrical current is a major safety concern.

Shannon derived an equation that describes the boundary between damaging and non-damaging levels of charge density stimulation. For applications such as brain stimulation, the recommended safety limit for the charge density of a stimulation pulse is 30 µC/cm^2^. More recent studies that consider the effects of duty cycle, charge per phase, and charge density suggest safety limits of 0.5 µC per phase and 8 µC/cm², respectively^45^. The charge per phase and the charge density of the proposed BCC system are ~5 pC and ~6 × 10^-4^ µC/cm^2^, respectively, which are well below the safety threshold.

### Event-based encoding and packetization with SODA

With the recent high-channel-count MEAs, the large volume of recorded data presents significant challenges in terms of data transfer, power consumption, and safety (i.e., SAR, charge balance, etc.). SODA addresses these issues by reducing data from a large number of channels, thus leading to a reduced duty cycle. As illustrated in Fig. 2, SODA comprises multiple Δ modulated ADC and an event-based packetizer utilizing a ternary address-event representation (AER) packet protocol^27^, which is well-suited for energy-efficient serial BCC data transmission. Each Δ modulated ADC is time-multiplexing to sample 16 analog input channels.

The Δ modulated ADC performs event-based sampling at 7-bit resolution and outputs data only when variations in the input signal exceed a predefined amplitude threshold, and it remains inactive during periods of negligible or no signal activity, without significant signal degradation.

The encoding step size of the Δ-modulator is programmable, allowing the system to adapt to different MEA characteristics and recording conditions. For example, it can be configured to have a larger encoding step when spike amplitudes are high to achieve a better compression ratio (i.e., lower duty cycle in BCC transmission). For a 7-bit Δ-modulated ADC, the normalized root-mean-square error (NRMSE) between the original dataset and the reconstructed signals is within 8.2%, corresponding to an SNR of > 21.7 dB.

**Fig. 3:**
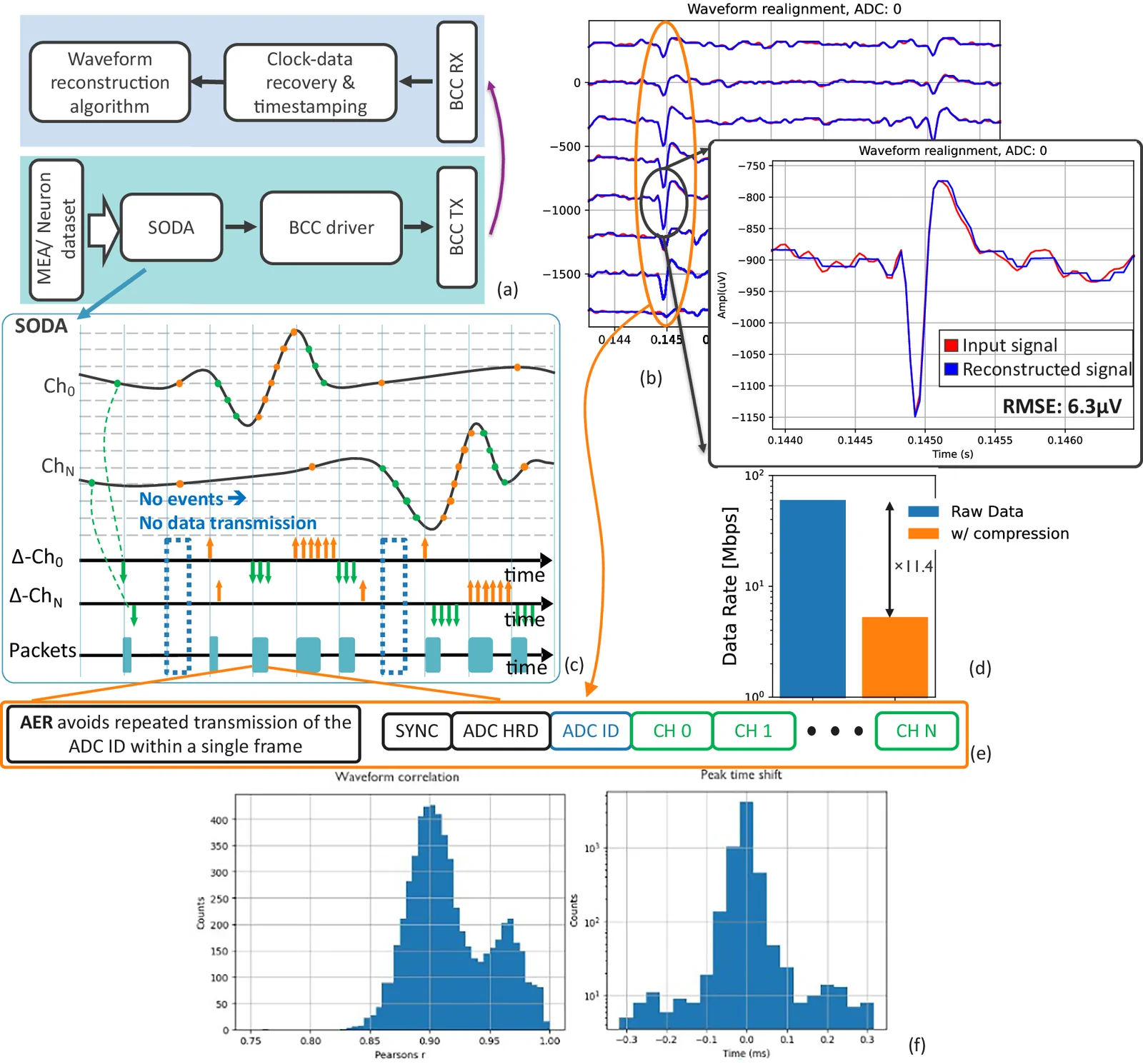
Send-on-delta encoder (SODA). **a** The SODA digitizes the input signal using Δ modulation and implements an event-based packetizer that employs a ternary address-event representation (AER) packet protocol, suited for energy-efficient serial data transmission. On the receiver side, clock and data are recovered together with the timestamping information. **b** Input signal waveform (red) and reconstructed waveform (blue). **c** SODA implements a Δ modulation and an event-based packetizer that transmits data packets only during neural activity, remaining silent in the case of the absence of events. **d** SODA achieves 7-12× compression rate. **e** AER bundles events from multiple channels into a packet and incorporates a spatial grouping technique. **f** Waveform correlation and spike timing shift histogram between original and reconstructed signals.

The proposed packetized ternary AER bundles events from multiple channels into a serial ternary-coded packet for data transmission and incorporates a spatial grouping technique to further reduce the protocol overhead, as shown in Fig. 3e.

In high-density MEA probes, a spike fired by a single neuron can be recorded simultaneously by several adjacent channels. As a result, events from neighbouring channels exhibit strong spatial correlation, allowing time-multiplexing of adjacent channels into a single ADC, as shown in Fig. 3b. By leveraging this spatial correlation, the proposed AER scheme avoids repeatedly transmitting the same ADC identifier for every event. Instead, the ADC ID is transmitted only once per packet for each ADC group. And to indicate the beginning of each ADC group, an ADC header (ADC HDR) is inserted into the packet.

The output packet consists of a sequence of synchronization (SYNC) bits, followed by the ADC header (ADC HDR), the ADC address (ADC ID), and one or more channel identification words (CH ID) associated with the same ADC. Each CH ID contains a 1-bit sign (SIGN) and a 4-bit channel address, and it may be repeated multiple times depending on the quantized delta (“Δ” value). This spatial grouping approach can reduce protocol overhead by up to 2× compared to the conventional serial AER without spatial grouping^46^.

On the receiving end, an event-based deserializer is implemented on an FPGA to recover the clock and data from the subdural unit, which is subject to clock frequency variations of up to 10%, as shown in Fig. 3a.

We evaluated the performance of the SODA using a public pre-recorded neural dataset from the^47^ database as input. The dataset was loaded into the Δ modulator, and the output data was reconstructed using an accumulation function. The reconstructed waveform demonstrates a high-fidelity match between the reconstructed waveform and the original pre-recorded signal, with an RMS error (RMSE) lower than 6.3 µVrms, as shown in Fig. 3b.

The SODA has also been evaluated with the in-vivo dataset^47^. The results show that 97.71% of spikes were “recovered”, with 4.15% false positives and 2.29% false negatives among a total of 6074 spikes. This corresponds to a precision of 96.0% and a recall of 97.7%. The spikes are sorted based on the standard sorting tool Kilosort. The recovery criteria are defined: (1) the waveform correlation is higher than 0.8, (2) the spike timing shift is less than 0.3 ms. The waveform correlation and spike timing shift histogram between the reconstructed spikes and original data are shown in Fig. 3f.

The AER packetizer, and the high compression ratio achieved by the Δ modulator significantly reduce the average transmission bit rate. This compression facilitates duty-cycled data transmission, allowing for the periodic activation of the charge redistribution system in the BCC transmitter. During non-transmission intervals, this system redistributes accumulated charge on the dipole coupler, thereby reducing both charge buildup and the overall transmission power density. The overall compression ratio, including additional timestamping information, was measured to be 7–12×, indicating that the same amount of information can be transmitted with a duty cycle down to ~13%. This approach reduces the average power absorbed by tissue, thereby increasing the margin to fulfil SAR requirements. These effects contribute to an improved safety margin without negatively affecting the signal reconstruction quality.

### Experimental results: Studying BCC interaction with neuronal network/brain-on-a-chip cultures

To evaluate the interaction and the safety of the proposed communication system with neural tissue, we conducted experiments using in vitro grown neuronal networks. We aimed to assess whether neural activities in the neural network change with and without active BCC transmission. Given that both neurons and BCC devices utilize electrical currents to transfer information, it is essential to assess the potential risk of unintended neuronal stimulation by the communication device. Furthermore, to our knowledge, no existing research on BCC for BCI applications directly addresses this safety concern. The selection of the frequency band is influenced by both the types of biological tissues that the signal must propagate through, as well as their geometric configurations. Choosing an appropriate signal bandwidth is critical for maximizing transmission speed, minimizing power consumption, and preventing unintended neurostimulation.

The frequency band between BCC signals (from 10’s to 100’s of MHz) and the typical neural stimulation signals (from 10’s to 100’s kHz)^48^ is different, and there remains a lack of conclusive studies demonstrating that BCC at these frequencies does not interfere with normal neuronal activity. To the best of the authors’ knowledge, there is no clear evidence in the literature indicating that the neurons are unaffected by high-frequency signals >10 MHz. Additionally, existing simulation software and the leaky integrate-and-fire models of neurons are not designed to reliably account for the high frequencies used in BCC. To empirically assess the potential correlation between BCC and neuronal activity, we conducted an experiment involving brain organoids and BCC TX devices.

**Fig. 4:**
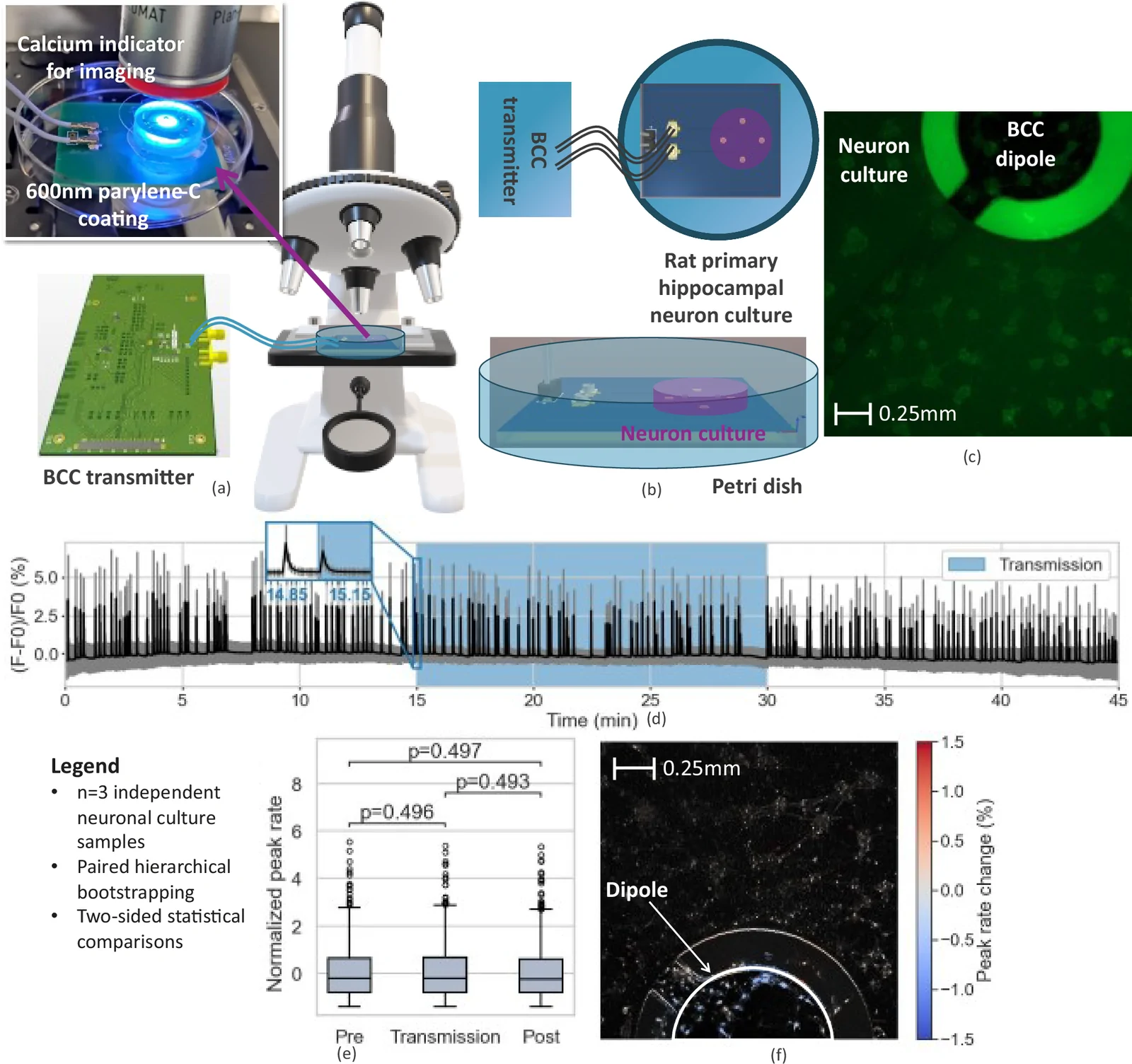
BCC interaction with in vitro grown neuronal cultures. **a** Experimental setup for calcium indicator imaging. The samples are placed under a microscope within a temperature- and humidity-controlled chamber. A custom BCC transmitter and culture board are used to simulate data transmission. **b**, The culture board contains both the neuronal culture, cultivated in an insert, and the dipole couplers used for BCC. Each board is coated with a 600 nm layer of parylene-C via atomic vapor deposition. **c** The cultivated neuronal culture near a dipole coupler. **d** Measurement results from calcium imaging, starting with a baseline of 15 minutes recording, then a 15-minute recording during transmission, followed by a 15-minute recording without transmission. **e**, The normalized peak rate to the peak rate of the baseline. **f** Spatial distribution of peak rate changes indicates that the inhibitory effect is localized only on the region above the dipole surface during prolonged transmission times of 15 minutes or longer.

For the experiment, a BCC transmitter device has been fabricated, as shown in Fig. 4a. While the BCC ASIC is placed on the BCC-TX board, the dipole couplers are situated on the culture board (Fig. 4b), where the neural cells are cultured according to the protocol detailed in the methods section. To ensure culture viability and enhance signal integrity, the culture board has been encapsulated with a thin layer of parylene-C using a vapor deposition process, resulting in a coating layer of 0.6 μm.

Primary hippocampal neurons from rat brains, sourced from Lonza^49^ were cultured on top of the culture board, and more specifically on top of and near the dipole coupler. Neurons were cultured for three weeks prior to calcium imaging using either Cal250, a chemical calcium indicator, or GCaMP, a genetically encoded calcium indicator.

Calcium imaging is used to monitor intracellular calcium levels in live cells, particularly to measure calcium transients associated with neural activity. This technique enables the recording of neuronal activities without relying on electrical measurements, as in electrophysiological methods, thereby minimizing the risk of interference from BCC signals. Performing the experiment on neuronal cultures allows for direct contact between the neurons and the surface of the dipole coupler, as shown in Fig. 4c. While this condition cannot be replicated in real physiological communication, where the dipole coupler would face the dura mater, we designed the setup to simulate the worst-case yet practical scenario where the dipole faces the neurons.

The experiment has been conducted on six samples in a temperature and humidity-controlled chamber that maintains constant environmental conditions. The objective of the experiment was to determine whether BCC communication induces any unintended alterations in the natural neural activity of brain cultures. With each culture demonstrating a slight variation on the baseline firing pattern, a baseline must be recorded to verify the spontaneous firing rate of each culture. The experiment proceeded by activating the BCC, which transmitted a data packet signal with a ~ 20% duty cycle for a defined period, after which the BCC was disabled. Throughout the experiment, calcium imaging was used to continuously monitor the firing patterns of the neurons. Measurements were performed on each sample for one to two days.

The study results, as shown in Fig. 4d, display fluorescence intensity on the y-axis and time on the x-axis. Each peak in intensity corresponds to a calcium peak generated by the neurons. The recorded peak rate was normalized to the baseline peak rate, as shown in Fig. 4e. The graph doesn’t show a significant variation in normalized peak rate across the pre-communication, during-communication, and post-communication periods. Further analysis of the spatial distribution of calcium peak rates suggests a reduction in the number of calcium peaks occurring only during prolonged transmission times of 15 min or longer. This reduction may be caused by a localized increase in temperature, as it is well known that heating can induce inhibition. This effect is more clearly represented in the spatial distribution plot of the peak rate changes shown in Fig. 4f, which reveals an inhibitory response of the neuronal cultures that is localized only to the region directly above the dipole surface. Notably, this inhibitory effect is absent in the immediate vicinity of the dipole, indicating that the phenomenon is highly restricted to the dipole surface itself. This interaction is only possible due to the experimental condition in which the neuronal cell culture is in direct contact with the transmitter dipole, which is equivalent to positioning a subdural unit with its dipole facing the cortex. In practical applications, however, the dipole of a subdural unit faces the dura, which would limit this inhibitory effect due to the distance of the neurons to the dipole surface.

Further studies are necessary to comprehensively rule out any potential adverse effects. Continued investigation is essential to ensure long-term safety and reliability of the system.

### Experimental results: Ex vivo validation with cadaver head specimens

**Fig. 5:**
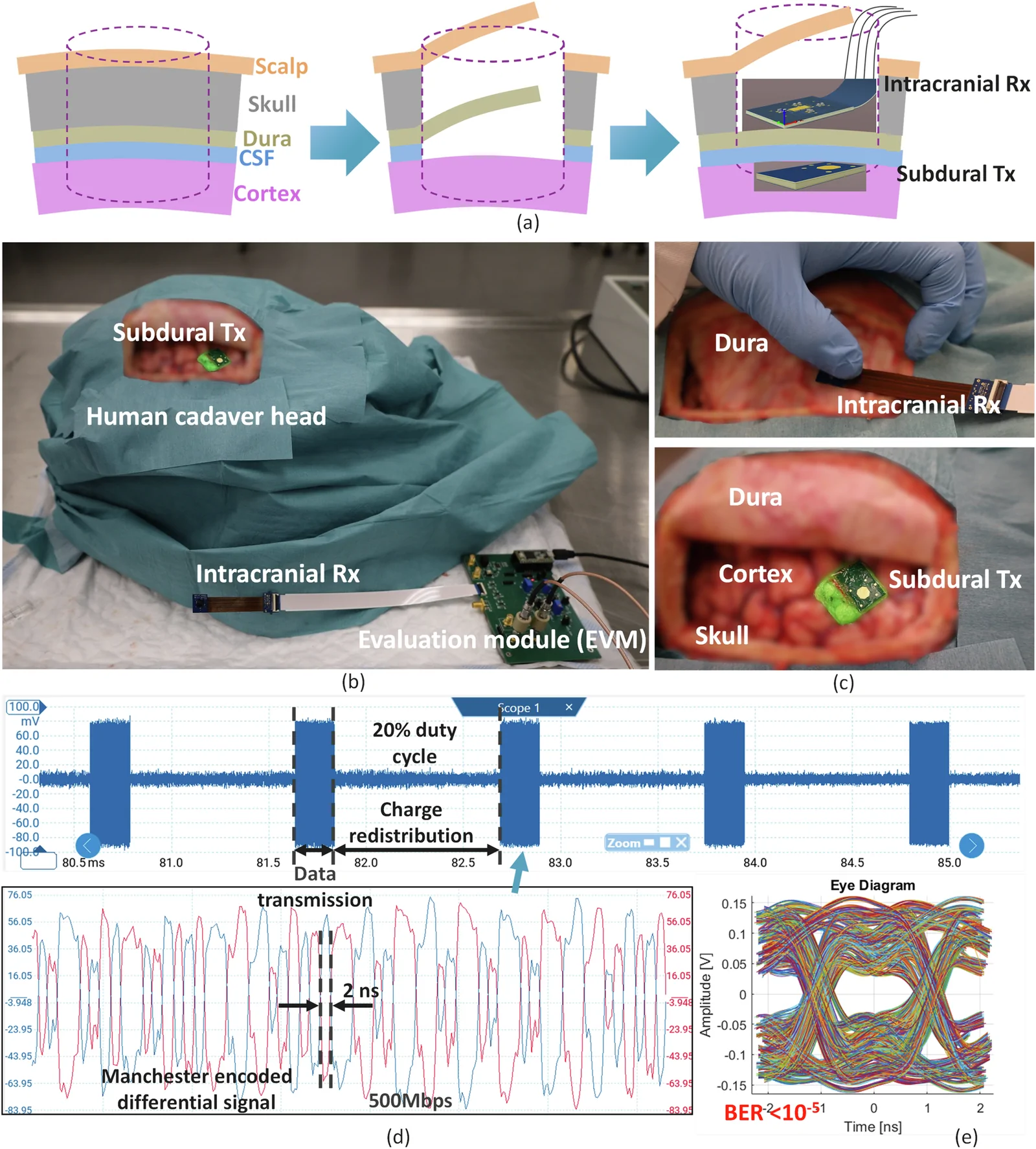
BCC validation with an ex vivo experiment using a human head cadaver specimen. **a** Procedure for the preparation of the specimen. After the incision, the subdural Tx is positioned on the cortex, the dura mater is placed in its original position, and the intracranial Rx is positioned above the dura. **b** A photograph of the experimental setup is shown, including the prototype transceiver. **c** The subdural Tx and intracranial Rx are positioned at their respective locations within the specimen. **d** The measurement results of the received signal at 500 MHz with a duty cycle of 20%, allowing the remaining 80% period for the activation of the charge redistribution circuit. The signal is encoded using differential Manchester coding. **e** The eye diagram of the received signal. The communication achieved BER < 10^-5^.

An ex vivo experiment was conducted on a human cadaveric head specimen to validate the event-based transdural BCC. The cadaver head specimen was prepared for the experiment as illustrated in Fig. 5. For this validation, a large craniotomy was made. Next, an opening in the dura mater layer surrounding the brain was precisely made without damaging the cortex. The dura layer was then lifted, and the subdural unit was positioned on the cortical surface (Fig. 5b), after which the dura was repositioned to its original location. The intracranial unit was placed on top of the dura mater and connected to the EVM for relaying data to PC (Fig. 5c). To better mimic the presence of cerebral spinal fluid, saline solution was applied to maintain electrical conductivity and keep the tissue moist.

Synthetic data in the subdural unit is generated on-chip, which is processed by the SODA and transmitted via BCC through the dura mater at 500 MHz. The data received were collected by the EVM. The SODA generates data packets with a 20% duty cycle, allowing the charge redistribution system to discharge residual charge on the dipole coupler during the off-period and thereby prevent charge accumulation.

The data is Manchester encoded prior to transmission through the BCC transmitter to ensure DC-balanced signaling, and the module allows adjustment of the duty-cycle frequency. The biphasic modulation forces each bit to be followed by a bit of opposite polarity, resulting in a 500 MHz data-rate transmission with zero-mean voltage.

The subdural unit is enclosed in a custom-designed casing that protects the electronic components and securely houses the battery, and the subdural and intracranial units are encapsulated with parylene-C for biocompatibility and protection.

The experimental results are presented in Fig. 5d. Duty-cycled data packets are transmitted by the subdural unit through the dura mater and received by the intracranial unit. There is no charge accumulation observed on the received signal, suggesting the effectiveness of both the event-based transmission and the active charge-balancing circuitry. The received signal exhibits a calculated signal-to-noise ratio (SNR) of ~9.6 dB, given the bit error rate (BER) of below 10^-5^.

These results demonstrate that high-bandwidth data can be successfully transmitted through the dura mater and received by the neural hub, thereby validating the feasibility of the communication approach.

The ex-vivo experiment performed on a human cadaver head specimen serves as a preliminary demonstration of the proposed iBCI telemetry system. As such, several limitations should be acknowledged when comparing these results with in vivo conditions. In particular, the experiment does not replicate the presence of physiological cerebrospinal fluid (CSF), normal fluid circulation (blood and CSF), or the effects associated with chronic implantation. These factors limit the extent to which the present study can fully represent in vivo operation. The experiment provides an important initial validation of the proposed telemetry approach. Further studies, including in vivo and long-term evaluations, are required to support the deployment of this technology.

## Discussion

The presented rigid transdural PCB represents a first prototype developed to validate the concept of a dual-stage, free-floating iBCI system. The proposed architecture is not limited to a rigid PCB implementation and can be extended to flexible PCB technologies that better conform to biological tissue. Also, as a proof-of-concept prototype, the present design has not yet been optimized for size, and further miniaturization is expected, particularly with the future integration of a wireless power delivery system.

Although Manchester-coded biphasic signal transmission suppresses DC components, duty-cycle modulation may still introduce envelope-related artifacts that could interfere with neural recordings. In practice, however, these artifacts can be reduced through filtering or mean subtraction, both of which are commonly used in neural front-end systems.

The fibrotic encapsulation on overall system performance was not investigated in the present study and remains an important subject for future work.

In the presented work, a preliminary biological safety study has been conducted, which shows a non-significant neuron-galvanic BCC interaction. However, further investigation is necessary to study the localized inhibition effect observed on the electrodes and to assess the long-term safety of the proposed BCC communication system.

## Conclusions

This work presents a novel two-stage wireless architecture for distributed iBCI. By introducing a miniaturized, wideband, galvanic-coupled BCC telemetry system for transdural data transfer, we demonstrate a viable solution that offers high data rate, energy efficiency, and miniaturization while maintaining the MEA free-floating, which could potentially minimize scar tissue formation.

The integration of the Send-on-delta encoder (SODA) enables significant data compression of 7-12× and duty-cycled operation, reducing power consumption and thermal load in compliance with SAR safety limits. Phantom and ex vivo validation on a human cadaveric specimen, confirms the feasibility of achieving up to 500 Mbps transmission with 20% duty cycling and BER < 10^-5^. Additionally, experiments with brain cultures were conducted to evaluate the potential interaction of BCC signals with surrounding neural tissue and to identify any possible adverse effects. The results show an inhibitory neuronal response of the culture located in the region directly above the dipole coupler. In practical applications, however, the dipole coupler faces the dura mater as in Fig. 5b, c, which is expected to limit this inhibitory effect.

These findings demonstrate that the proposed BCC telemetry system offers a scalable, energy-efficient platform for next-generation, high-resolution neural interfaces intended for chronic deployment in neuroprosthetic applications.

## Methods

### BCC interaction with the neuronal networks

#### Brain on chips device design

For experimental purposes, a custom-designed culture board was fabricated to accommodate a dipole coupler. This board underwent an initial coating with a 0.6 µm layer of parylene-C, which was applied to ensure a non-reactive surface compatible with neuron cultures. This treatment allows direct neuron interaction with the surface without compromising cell viability.

The transmitter ASIC utilized in this setup generates a signal via an onboard 9-bit pseudo-random binary sequence (PRBS), simulating the transmission of recorded neural signals. The board is connected to a notebook, which supplies the necessary control signals and power to regulate its function.

To localize the cultured brain tissue within a defined region on the board, the setup incorporated a customized silicone two-well culture insert (Ibidi GmbH). This insert confines the neuronal culture to a specified area, facilitating consistent imaging and observation. To further enhance the setup’s durability and prevent any potential leakage of the culture medium, a supplementary epoxy ring (LOCTITE EA M-31CL) was applied around the culture area, ensuring a liquid-tight seal. The epoxy is then cured at 65 °C for 2 h. This preparation of the experimental environment is vital for maintaining stable experimental analysis conditions.

#### Cell cultures

The chip preparation and cell plating process began with a thorough cleaning of the chips in a 1% Tergazyme solution in H₂O, allowing them to soak overnight. The following day, chips were extensively rinsed with running high-purity water (HPW), then sprayed with 70% ethanol (EtOH) and air-dried within a biosafety cabinet. The chips were then handled exclusively under sterile conditions.

The chip’s active area was coated with 200 μL of poly-D-lysine (PDL) solution and incubated overnight to promote cellular adhesion. The next day, the PDL solution was removed, and the surface was rinsed three times with sterile HPW.

Rat hippocampal neurons (RHNs), sourced from Lonza, were defrosted following the standard protocol provided by the supplier. Briefly, the cell vial was defrosted in a warm water bath, and the cell suspension was transferred to a 50 mL conical Falcon tube, where 10 mL of culture medium was gradually added. Cells were then counted, centrifuged at 300 rcf for 5 min, and resuspended in a plating medium to the desired concentration. For optimal viability, B-27™ Plus Neuronal Culture System (Thermofisher Scientific) served as the primary medium, while the plating medium was supplemented with 20 μg/mL mouse laminin (Sigma-Aldrich) and 10 μM RHO/ROCK pathway inhibitor, Y-27632 (Stemcell technologies).

We used a high seeding density to promote network activity to enable a clear visualization of any alterations in this activity. The seeding density was 200,000 cells/cm², which equated to 200 μL of cell suspension for the chip’s surface area of approximately 0.5 cm² (cell suspension concentration: 5 × 10⁵ cells/mL in plating medium). Once plated, chips were carefully transferred to a cell culture incubator within custom humidity chambers to minimize evaporation.

On the following day, the plating medium was replaced with culture medium, filling each chip chamber. Thereafter, approximately 80% of the culture medium was refreshed every other day to maintain optimal neuronal culture conditions.

#### Transmission effect setup

The experiments were conducted using four brain culture samples, as shown in Fig. 4. Each session included baseline recordings with telemetry disabled, as well as recordings obtained during active communication.

Microscopy experiments were performed in a tightly regulated, temperature-controlled environment to maintain physiological conditions suitable for neural cells. A stable temperature of 37 °C was ensured using an Okolab temperature controller, which provided precise thermal regulation throughout the imaging sessions. Prior to imaging, the neuronal culture medium was partially replaced with BrainPhys medium to enhance optical clarity and improve image fidelity, thereby facilitating better visualization of neural structures and activity.

Neuronal activity imaging was performed using an upright widefield microscope (Axio Examiner, Zeiss) equipped with a 5x objective lens and configured to capture images at a 0.5 s frame rate. This setup allowed for detailed observation of dynamic neural responses in real-time. The BCC transmitter, incorporating an on-chip 9-bit pseudo-random binary sequence (PRBS) generator, was employed to simulate the consistent bitstream of data transmission in a neural implant context.

It is noteworthy that calcium imaging, the technique employed here to visualize neural activity, does not depend on electrical signals to monitor cellular processes. This characteristic of calcium imaging distinguishes it from methods like microelectrode array (MEA) recording, which directly detects electrical activity and is therefore susceptible to electromagnetic interference. In contrast, calcium imaging remains unaffected by the electrical emissions of the BCC transmitter, thereby offering a robust approach for capturing precise and interference-free measurements of neural activity.

### Ex vivo validation on cadaver specimens

#### Subdural unit design

The subdural unit initiates the configuration process by loading the IC’s configuration registers with the appropriate settings, including parameters such as clock frequency and communication protocols.

Once the configuration is complete, the µC transitions into a low-energy mode to save energy. This low-power state is maintained until a reset input signal is received, which triggers the µC to restart the configuration process.

An STM32 µC was selected for its low power consumption and compact form factor. The reset mechanism employs a Hall-effect diode switch, which can be externally triggered using a magnet. When a magnet is brought into proximity with the implant for approximately 10 s, the diode generates a reset signal, eliminating the need for direct physical contact with the device.

The subdural unit is enclosed in a custom housing measuring 19.4 × 21.5 mm², which also accommodates two LR60 button cell batteries.

The transcranial unit receives data transmitted by the subdural unit, whose configuration is initiated by the EVM. The EVM also supplies power and records the received data through an oscilloscope.

#### Experimental setup

Figure 5b, c shows the setup and the method for validation of the iBCI on a cadaver head specimen.

The specimen was prepared on the same day as the experiment. An incision was made on the scalp, followed by removal of the skull. Subsequently, an opening was created in the dura mater to expose the cortical surface.

The subdural unit was then positioned directly on the cortex, after which the dura mater was carefully repositioned. The transcranial unit was placed on top of the dura mater and connected to an oscilloscope via the EVM, allowing for the acquisition of transmitted data.

Throughout the procedure, tissue moisture level was maintained by regularly applying a saline solution to the specimen.

## Data Availability

The data that support the plots within this paper and other findings of this study are available from the corresponding author upon reasonable request.
